# Evaluation of the Performance of the Distributed Phased-MIMO Sonar

**DOI:** 10.3390/s17010133

**Published:** 2017-01-11

**Authors:** Xiang Pan, Jingning Jiang, Nan Wang

**Affiliations:** College of Information and Electronic Engineering, Zhejiang University, Hangzhou 310027, China; jakejiangjn@zju.edu.cn (J.J.); tingfengyu@zju.edu.cn (N.W.)

**Keywords:** broadband signal, distributed phased-MIMO, target and localization, sonar, experimental evaluation

## Abstract

A broadband signal model is proposed for a distributed multiple-input multiple-output (MIMO) sonar system consisting of two transmitters and a receiving linear array. Transmitters are widely separated to illuminate the different aspects of an extended target of interest. The beamforming technique is utilized at the reception ends for enhancement of weak target echoes. A MIMO detector is designed with the estimated target position parameters within the general likelihood rate test (GLRT) framework. For the high signal-to-noise ratio case, the detection performance of the MIMO system is better than that of the phased-array system in the numerical simulations and the tank experiments. The robustness of the distributed phased-MIMO sonar system is further demonstrated in localization of a target in at-lake experiments.

## 1. Introduction

Inspired by the fast development of multiple-input-multiple-output (MIMO) radar [1], we attempt to exploit the diversity gain for improvement of sonar performance. According to the array configurations, there are two kinds of MIMO systems, namely the distributed MIMO system [2] and the co-located MIMO system [3]. The distributed MIMO system may be regarded as one kind of multistatic system with widely-separated transmitters and receivers to capture the spatial diversity for enhancement of detection of an extended target. Meanwhile, the latter using the co-located transmitters and receivers enjoys the benefits from the waveform diversity for high resolution sensing of a point target. In this paper, we focus on the combination of the distributed MIMO system with the phased-array system, namely the distributed phased-MIMO sonar system, which can share both the diversity gain and the array gain for enhancement of robustness of target detection.

Since MIMO radar concept was firstly proposed in 2004 [1], many more achievements on the distributed MIMO system have been realized, such as improvement of the spatial resolution of targets [4] and the accuracy of target parameter estimation [5,6,7,8]. In [5], the transmitting array gain is combined with the target diversity for estimating target bearing. The distributed MIMO radar exploits knowledge of the phase differences measured at the receive antennas to produce a high-accuracy target location estimate [6]. The Cramer-Rao lower bound (CRLB) is derived for the target localization accuracy attainable by the use of the MIMO radar systems [7]. Symmetrically placing all the transmitters and all the receivers gives the best achievable target velocity estimation [8]. For the MIMO detection case, the optimal detector is derived in the Neyman-Pearson sense [9]. The diversity gain is discussed when the transmitted waveforms are non-orthogonal [10] as well as the target scattering coefficients follow non-Gaussian distribution [10,11]. The generalized likelihood ratio test (GLRT) detectors are respectively derived in homogeneous and nonhomogeneous clutter [12,13]. In [14], a detector within the GLRT framework is designed for the MIMO radar in the compound-Gaussian clutter. The joint target detection and time-delay estimation in the MIMO radar exhibit significant gains over the phased-array radar in detection of the extended targets [15]. In MIMO tracking, the Kalman filter (KF) outperforms the particle filter (PF) for the high SNR case [16]. High precision localization can be achieved by data confusion of the parameters estimated at each receiver [17]. The MIMO radar outperforms the multistatic system in multitarget tracking [18]. In [19], compressive sensing (CS) is utilized in a MIMO radar system for estimating the positions and velocities of multiple targets. A sparsity based detector developed for a moving platform performs better than the covariance matrix based detector in moving target detection [20].

Although a common MIMO processing framework is proposed for radar and sonar [21], the MIMO sonar technique develops slowly due to the large time-delay spread and the large Doppler shift of propagation in underwater acoustic channels, resulting in increasing of coherence of the orthogonal echoes. Thus, the orthogonal waveform design attracts much attention in the MIMO sonar, such as the phase-code sequence [22], the orthogonal frequency division signal and the spread-spectrum code [23]. A formulation with the clear separation of propagation and target reflection is presented for the broadband MIMO sonar systems [24]. With the spatial diversity, the MIMO sonar can produce a higher resolution image than the synthetic aperture sonar (SAS) using the same frequency band and at the same range [25]. In addition, the MIMO sonar has to deal with omnidirectional transmission, resulting in an increase of the level of reverberation in shallow water environments.

The main contributions of the paper include: (1) A joint processing framework with the broad-band signal model has been proposed for the distributed phased-MIMO sonar system. Due to enjoying the spatial diversity gain and the array gain, the distributed phased-MIMO sonar system performs better than the traditional phased-array sonar system in localization of a target in at-lake experiments; (2) Both target bearing and target range can be estimated by the distributed phased-MIMO sonar system. With the estimated target position parameters, the designed generalized likelihood ratio test (GLRT) detector performs well in numerical simulations. In the related literature, the existing MIMO detectors break the space into small cells and detect the presence of the target in a specific cell [3,9], or only estimate the time-delay parameter in the GLRT framework [15]. To our knowledge, there is only one similar article to be published which is related with the collocated phased-MIMO radar system [26].

The rest of the paper is organized as follows. The distributed phased-MIMO sonar processing framework is proposed in Section 2. With the estimated target position parameters, the GLRT detector is respectively derived for the narrow band and broadband signals. Section 3 evaluates the performance of the distributed phased-MIMO sonar system using numerical simulations. Section 4 demonstrates the robustness of the distributed phased-MIMO sonar system by localization of a target in a shallow water environment. Some conclusions are drawn in Section 5.

## 2. Distributed Phased-MIMO Sonar Processing Framework

### 2.1. Narrowband Signal Model

In the far field, we assume that an extended target is modeled as a short bar with length of D. We consider a distributed phased-MIMO sonar system consisting of M transmitting transducers and N hydrophones. These transducers are widely separated to see different aspects of the target. In contrast, N hydrophones are placed closely to enable direction measurement. For simplicity, these hydrophones are assumed to be laid out as a linear array with the uniform spacing of $d_{r}$. In order to capture the target diversity, the array configuration of the distributed phased-MIMO sonar system meets the following constraint condition [5]:

(1) $$d_{t}\geq \frac{\lambda R}{D}$$

where $d_{t}$ denotes the inter-element spacing between two adjacent transducers, λ is wavelength of the transmitted signal, and R is distance from the center of two transducers to the target. There is an underlying physical interpretation for Equation (1) [9]. The target is illuminated by the transmitting transducer and it reflects the energy back. The extended target can be regarded as a receiving array with aperture D and beamwidth λ/D. If two transmitting transducers are not within the same receiving beamwidth of the target, then they can see different aspects of the target.

With the previous assumptions, M waveforms are simultaneously transmitted to illuminate the target from different angles, the echo received by the ith (i=1,⋯,N) element of the receiving array can be expressed as

(2) $$\begin{array}{cc} r_{i}(t,\theta ) & =\sqrt{E/M}\sum_{k=1}^{M}\sum_{q=1}^{Q}\varsigma_{q}e^{-j2\pi f_{k}\tau_{ik}^{q}}s_{k}(t-\tau_{ik})+w_{i}(t)\\ & =\sqrt{E/M}\sum_{k=1}^{M}h_{ik}s_{k}(t-\tau_{1k})e^{-j2\pi (i-1)d_{r}\sin(\theta )/\lambda_{k}}+w_{i}(t) \end{array}$$

where $\varsigma_{q}$ denotes the complex reflectivity coefficient of the qth (q=1,⋯,Q) scatterer, $2\pi f_{k}\tau_{ik}^{q}$ represents the effect of propagation phase shift due to the qth scatterer, $\tau_{ik}$ denotes the time-delay from the kth transmitter to the ith receiver, $\sqrt{E/M}$ denotes the total transmitted power to be evenly shared by all transmitters, and $w_{i}(t)$ contains reverberation and noise. For simplicity, we define $h_{ik}=\sum_{p=1}^{P}\varsigma_{p}e^{-j2\pi f_{k}\tau_{ik}^{p}}$ as the propagation channel among the kth transmitter, the target, and the ith receiver. $\tau_{1k}$ is the time propagating from the kth transmitter to the target and back to the first element of the receiving array. $2\pi (i-1)d_{r}\sin(\theta )/\lambda_{k}$ presents the phase-difference between echoes received at two positions, θ denotes the direction of arrival (DOA) of the reflected waves, $\lambda_{k}$ is wavelength of the kth transmitted waveform $s_{k}(t)$, $\int_{T_{t}}s_{k}(t)s_{l}^{*}(t)dt=\delta (k-l)$ (k,l=1,⋯,M), $T_{t}$ denotes duration of the transmitted signal. Due to underwater acoustic multipath propagation resulting in the increasing of correlation between orthogonal signals in the time domain, signals without overlap in the frequency domain are chosen as transmitted orthogonal waveforms in the experiments. It is noted that we neglect the path-losses and the absorptive attenuation in Equation (2). Different from those of [3,9,15], the model in Equation (2) contains the DOA parameter θ.

Assume that the received signals are sampled at the rate of $1/T_{s}$, and we define $r_{i}^{k}(l;\theta )=r_{i}^{k}(lT_{s},\theta )$, (l=0,⋯,L−1). After target echoes are separated using a matched filtering bank, the echo corresponding to the kth transmitted signal can be expressed as

(3) $$r_{i}^{k}(l;\theta )=\sqrt{E/M}h_{ik}s_{k}(l;\tau_{ik})e^{-j2\pi (i-1)d_{r}\sin(\theta )/\lambda_{k}}+w_{i}^{k}(l)$$

For avoiding the angle ambiguity in the DOA measurement, $d_{r}=\lambda_{\min}/2$ is chosen where $\lambda_{\min}$ is the shortest wavelength of the set $\lambda_{k}$ (k=1,⋯,M).

We assume that the multiple orthogonal waveforms are synchronized to illuminate the extended target and the corresponding echoes are received by the receiving array. The angle θ can be estimated at the receiving array by maximizing the term $\sum_{k=0}^{M-1}{\left|\sum_{l=0}^{L-1}a^{k}{\left(\theta^{\prime }\right)}^{H}r^{k}(l;\theta )\right|}^{2}$, the steering vector $a^{k}(\theta^{\prime })=[a_{1}^{k}(\theta^{\prime }),\cdots ,a_{N}^{k}(\theta^{\prime })]$, $a_{i}^{k}(\theta^{\prime })=\frac{1}{\sqrt{N}}e^{j2\pi (i-1)d_{r}\sin(\theta^{\prime })/\lambda_{k}}$, $r^{k}(l;\theta )=[r_{1}^{k}(l;\theta ),\cdots r_{N}^{k}(l;\theta )]$. Thus, the estimated DOA $\overset{⌢}{\theta }$ is

(4) $$\overset{⌢}{\theta }=\underset{\theta^{\prime }}{\mathrm{arg max}}\sum_{k=0}^{M-1}{\left|\sum_{l=0}^{L-1}a^{k}{\left(\theta^{\prime }\right)}^{H}r^{k}(l;\theta )\right|}^{2}$$

From Equation (4), we can see that it combines the array gain provided by beamforming operation with the target diversity exploited by transmitting multiple orthogonal signals.

With the estimated $\overset{⌢}{\theta }$, the enhanced target echo can be expressed as

(5) $$y^{k}(l)=a^{k}{\left(\overset{⌢}{\theta }\right)}^{H}r^{k}(l;\theta )$$

Correspondingly, the time-delay ${\overset{⌢}{\tau }}_{k}$ can be estimated by matched filtering the transmitted signal $s_{k}(l)$ with the enhanced target echo.

(6) $${\overset{⌢}{\tau }}_{k}=\underset{\tau }{\mathrm{arg max}}{\left|\sum_{l=0}^{L-1}s_{k}^{*}(l;\tau )y^{k}(l)\right|}^{2}$$

With the time-delay ${\overset{⌢}{\tau }}_{k}$, the target distance ${\overset{⌢}{d}}_{k}$ relative to the receiving array can be calculated by

(7) $${\overset{⌢}{d}}_{k}=\frac{{\left(c{\overset{⌢}{\tau }}_{k}\right)}^{2}-d_{\mathrm{tr}}^{k2}}{2c{\overset{⌢}{\tau }}_{k}+2d_{\mathrm{tr}}^{k}\cos(\overset{⌢}{\theta })}$$

where $d_{\mathrm{tr}}^{k}$ denotes distance between the kth transmitter and the phase center of the receiving array, and c is speed of sound in water.

With the estimated parameters $\left(\overset{⌢}{\theta },{\overset{⌢}{\tau }}_{k}\right)$, the GLRT detector in the Neyman-Pearson sense [27] can be given by

(8) $$T=\sum_{k=1}^{M}\sum_{i=1}^{N}{\left|\sum_{l=0}^{L-1}s_{k}^{*}(l;{\overset{⌢}{\tau }}_{k})r_{i}^{k}(l;\overset{⌢}{\theta })\right|}^{2}\overset{H_{1}}{\underset{H_{0}}{\underset{<}{>}}}\delta$$

where $H_{1}$ and $H_{0}$ respectively denote the presence of a target or no target. For simplicity, we assume the term $w_{i}(t)$ in Equation (2) is a zero-mean white Gaussian noise with variance $\sigma_{w}^{2}I_{MN}$ and $\varsigma_{p}$ is a complex random variable with variance 1/P. Correspondingly, $\beta_{ik}$ follows a zero-mean complex random variable with variance $I_{MN}$, and T is distributed as follows

(9) $$T\sim \{\begin{array}{ll} \frac{\sigma_{w}^{2}}{2}\chi_{2NM}^{2} & H_{0}\\ (\frac{E}{2M}+\frac{\sigma_{w}^{2}}{2})\chi_{2NM}^{2} & H_{1} \end{array}$$

where $\chi_{d}^{2}$ denotes a chi-square random variable with d degrees of freedom.

The probability of false alarm in a general case can be expressed as

(10) $$P_{fa}=\mathrm{Pr}(T>\delta |H_{0})=\mathrm{Pr}(\frac{\sigma_{w}^{2}}{2}\chi_{2NM}^{2}>\delta )=\mathrm{Pr}(\chi_{2NM}^{2}>\frac{2\delta }{\sigma_{w}^{2}})=1-F_{\chi_{2NM}^{2}}(\frac{2\delta }{\sigma_{w}^{2}})$$

(11) $$F_{\chi_{2NM}^{2}}(\frac{2\delta }{\sigma_{w}^{2}})=1-P_{fa}$$

where $F_{X}\left(*\right)$ denotes the cumulative distribution function of a random variable *X*. Correspondingly, the threshold δ is set using the following formula

(12) $$\delta =\frac{\sigma_{w}^{2}}{2}F_{\chi_{2NM}^{2}}^{-1}(1-P_{fa})$$

where $F_{\chi_{2NM}^{2}}^{-1}$ denotes the inverse cumulative distribution function of a chi-square random variable with 2NM degrees of freedom. The probability of detection is given by

(13) $$\begin{array}{l} P_{d}=\mathrm{Pr}(T>\delta |H_{1})=\mathrm{Pr}\left\{(\frac{E}{2M}+\frac{\sigma_{w}^{2}}{2})\chi_{2NM}^{2}>\delta \right\}\\ =1-F_{\chi_{2NM}^{2}}\left(\frac{2\delta }{\frac{E}{M}+\sigma_{w}^{2}}\right)\\ =1-F_{\chi_{2NM}^{2}}\left\{\frac{\sigma_{w}^{2}}{\frac{E}{M}+\sigma_{w}^{2}}F_{\chi_{2NM}^{2}}^{-1}(1-P_{fa})\right\} \end{array}$$

### 2.2. Broadband Signal Model

Due to the broadband signals providing higher resolution and being less sensitive to interference over the narrow band signals, we further discuss the broadband signal model for the distributed phased-MIMO sonar system. Assume that the whole frequency band of the transmitted signal is segmented into M non-overlap sub-bands, each sub-band with *L* frequency bins, and the kth sub-band is allocated to the kth transmitter. Thus Equation (2) is rewritten in the frequency domain as

(14) $$r_{i}(f_{l},\theta )=\sqrt{E/M}\sum_{k=1}^{M}h_{ik}^{l}s_{k}(f_{l})e^{-j2\pi f_{l}\tau_{1k}}e^{-j2\pi (i-1)d_{r}\sin(\theta )f_{l}/c}+w_{i}(f_{l})$$

where $h_{ik}^{l}$ denotes the propagation channel corresponding to the frequency bin $f_{l}$, and ${\left\|s_{k}(f_{l})\right\|}^{2}=1/L$ denotes that the power is distributed over the whole sub-band.

Correspondingly, the bearing of the target can be estimated by

(15) $$\overset{⌢}{\theta }=\underset{\theta^{\prime }}{\mathrm{arg max}}\sum_{k=0}^{M-1}{\left|\sum_{l=0}^{L-1}a^{k}{\left(\theta^{\prime }\right)}^{H}r^{k}(f_{l},\theta )\right|}^{2}$$

where $a^{k}(\theta^{\prime })=[a_{1}^{k}(\theta^{\prime }),\cdots ,a_{N}^{k}(\theta^{\prime })]$, $a_{i}^{k}(\theta^{\prime })=\frac{1}{\sqrt{N}}e^{j2\pi (i-1)d_{r}\sin(\theta^{\prime })f_{l}/c}$, $r^{k}(f_{l},\theta )=[r_{1}^{k}(f_{l},\theta ),\cdots r_{N}^{k}(f_{l},\theta )]$, $r_{i}^{k}(f_{l},\theta )=\sqrt{E/M}\beta_{ik}^{l}s_{k}(f_{l})e^{-j2\pi f_{l}\tau_{1k}}e^{-j2\pi (i-1)d_{r}\sin(\theta )f_{l}/c}+w_{i}(f_{l})$.

The time-delay ${\overset{⌢}{\tau }}_{k}$ can be estimated by

(16) $${\overset{⌢}{\tau }}_{k}=\underset{\tau }{\mathrm{arg max}}{\left|\sum_{l=0}^{L-1}s_{k}^{*}(f_{l})e^{j2\pi f_{l}\tau }y^{k}(f_{l})\right|}^{2}$$

where $y^{k}(f_{l})=a^{k}{\left(\overset{⌢}{\theta }\right)}^{H}r^{k}(f_{l},\theta )$. With the time-delay ${\overset{⌢}{\tau }}_{k}$, we can estimate the target range ${\overset{⌢}{d}}_{k}$ using Equation (7).

With the estimated parameters $\left(\overset{⌢}{\theta },{\overset{⌢}{\tau }}_{k}\right)$, the corresponding GLRT detector can be given by

(17) $$T=\sum_{k=1}^{M}\sum_{i=1}^{N}{\left|\sum_{l=0}^{L-1}s_{k}^{*}(f_{l})e^{j2\pi f_{l}{\overset{⌢}{\tau }}_{k}}r_{i}^{k}(f_{l},\overset{⌢}{\theta })\right|}^{2}\overset{H_{1}}{\underset{H_{0}}{\underset{<}{>}}}\delta$$

Assume that $w_{i}(f_{l})$ is a zero-mean complex Gaussian white noise with variance $\sigma_{w}^{2}I_{NML}$ and $\beta_{ik}$ is a zero-mean complex Gaussian white noise with variance $I_{NML}$. Thus, T is distributed as follows

(18) $$T\sim \{\begin{array}{cc} \frac{\sigma_{w}^{2}}{2}\chi_{2NML}^{2} & H_{0}\\ (\frac{E}{2ML}+\frac{\sigma_{w}^{2}}{2})\chi_{2NML}^{2} & H_{1} \end{array}$$

The probability of false alarm can be expressed as

(19) $$P_{fa}=\mathrm{Pr}(T>\delta {|H}_{0})=\mathrm{Pr}(T>\delta |H_{0})=\mathrm{Pr}\left(\chi_{2NML}^{2}>\frac{\delta }{\sigma_{w}^{2}}\right),$$

The threshold δ is set using the following formula

(20) $$\delta =\sigma_{w}^{2}F_{\chi_{2NML}^{2}}^{-1}(1-P_{fa})$$

The probability of detection is given by

(21) $$\begin{array}{l} P_{d}=\mathrm{Pr}(T>\delta |H_{1})=\mathrm{Pr}\left((\frac{E}{2ML}+\frac{\sigma_{w}^{2}}{2})\chi_{2NML}^{2}>\delta \right)\\ \;=1-F_{\chi_{2NML}^{2}}\left(\frac{\sigma_{w}^{2}}{\frac{E}{ML}+\sigma_{w}^{2}}F_{\chi_{2NML}^{2}}^{-1}(1-P_{fa})\right) \end{array}$$

### 2.3. MIMO Detection in Shallow Water Environment

In practice, reverberation is a challenging problem for the phased-MIMO sonar system operating in shallow water. The reverberation power is spread over the duration of the transmitted waveform. In addition, due to undergoing distortion during two-way propagation, namely forward propagation and backward scattering, the active sonar system cannot achieve the full performance using the coherent matched filter. For alleviating mismatch between the traditional matched filter and the propagation channel parameters, we replace the matched filter in the distributed phased-MIMO sonar system by the replica correlation integration (RCI) processor [28]. In the at-lake experiments of localization of a target in a shallow water environment, it is reasonable to model the multipath propagation channel as the time spreading distortion channel. Thus, Equation (6) is rewritten as

(22) $${\overset{⌢}{\tau }}_{k}=\underset{\tau }{\mathrm{arg max}}{\sum_{p=0}^{P-1}\left|\sum_{l=0}^{L-1}s_{k}^{*}(l;\tau +p)y^{k}(l)\right|}^{2}$$

where $P=T_{s}f_{s}$, $T_{s}$ denotes the effective length of the spreading waveform, and $f_{s}$ is the sampling rate.

Correspondingly, Equation (16) is rewritten as

(23) $${\overset{⌢}{\tau }}_{k}=\underset{\tau }{\mathrm{arg max}}{\sum_{p=0}^{P-1}\left|\sum_{l=0}^{L-1}s_{k}^{*}(f_{l})e^{j2\pi f_{l}(\tau +p)}y^{k}(f_{l})\right|}^{2}$$

## 3. Numerical Simulations

In this section, we design some simulation experiments to evaluate detection performance of the distributed phased-MIMO sonar system by comparing it with that of the traditional phased-array sonar system. We consider the phased-MIMO system having a nine-element receiving linear array and two transmitters, that is M=2, N=9. Two transmitters are located at two ends of the receiving array. For the phased-array sonar system, it consists of one transmitter and a nine-element linear array. The array configurations are similar to those utilized in the at-lake experiments. Two kinds of signals are considered in numerical simulations, 4 ms PCW signal with frequency of 7.5 kHz and 8.5 kHz, and 4 m LFM signals with frequency of 7–8 kHz and 8–9 kHz. The sampling rate is 48 kHz.

The receiving array has the inter-element spacing of 7.5 cm. Thus, it can avoid the angle ambiguity in the DOA estimation. Two transmitters are separated with distance of 2.4 m which meets the requirement of capturing the target diversity. Assume that the target is modeled as a short bar with length of 0.5 m and consists of 100 evenly-distributed scatterers, namely Q=100. The target is assumed to be placed at distance of 5 m and parallel to the receiving array. The speed of sound in water is assumed to be 1500 m/s.

**Figure 1:**
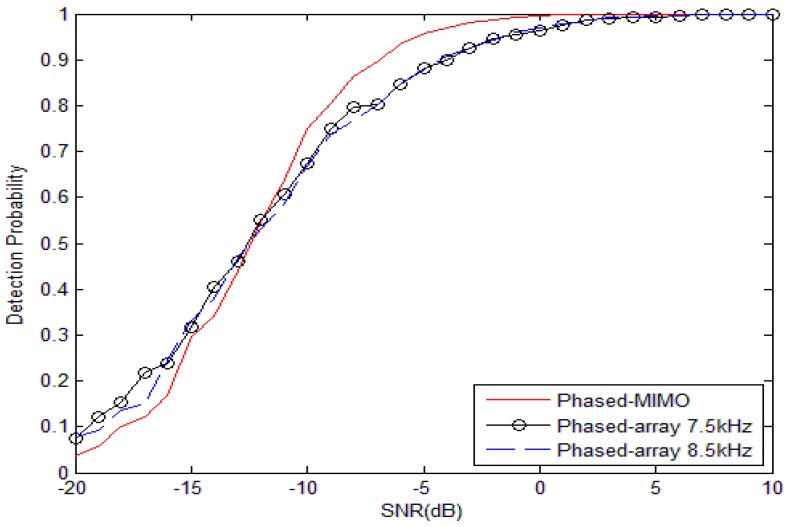
Probability of detection as a function of the SNR for the narrow band signal with frequency of 7.5 kHz and 8.5 kHz. The probability of false alarm is fixed at 0.001. M=2, N=9, Q=100.

Figure 1 depicts the probability of detection of the GLRT detector as the function of the SNR. The probability of false alarm is fixed at 0.001. At low SNR, the phased-array sonar system outperforms the distributed phased-MIMO sonar system. When SNR is higher than −11 dB, the distributed phased-MIMO sonar system performs better than the phased-array sonar system. For −6 dB, the distributed phased-MIMO system has the probability of detection of 93.43%, and the phased-array system has the probability of detection of 84.72% for 7.5 kHz and 84.78% for 8.5 kHz. The conclusion is consistent with that achieved in [3], namely the MIMO system having its superiority in the high SNR realm. Due to neglecting absorbing attenuation, there is almost the same performance for the phased-array sonar system with a frequency of 7.5 kHz and 8.5 kHz.

**Figure 2:**
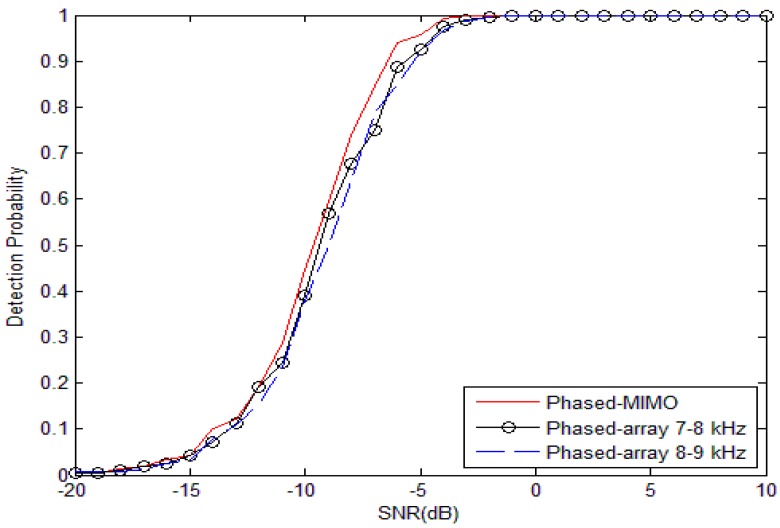
Probability of detection as a function of the SNR for the broad-band signal with frequency of 6–8 kHz and 8–10 kHz. The probability of false alarm is fixed at 0.001. M=2, N=9, Q=100.

For the broadband signal, the same conclusion can be drawn from Figure 2 where the distributed phased-MIMO sonar system performs better than the phase-array sonar system when SNR is larger than −19 dB. For −6 dB, the distributed phased-MIMO sonar system has the probability of detection of 93.9%, and the phased-array sonar system has the probability of detection of 88.71% for 7–8 kHz and 85.03% for 8–9 kHz. It is obvious that the distributed phased-MIMO sonar system with the broadband signal performs better than with the narrow band signal due to the diversity gain in the high SNR region. However, in the low SNR region, such as SNR = −10 dB, by comparing Figure 1 and Figure 2, the detection probability of the narrowband signal looks higher than that of the broadband signal. For the low SNR case, coherent processing plays a significant role over diversity processing. Thus, the phased-array system with a narrowband signal has better detection ability than with the broadband signal due to frequency diversity, but the latter has strong robustness against interference.

## 4. Experimental Results and Analysis

In this section, we design some experiments to test the effectiveness of the signal model of the distributed phased-MIMO sonar system. The detection performance is evaluated using the tank experimental data while the localization performance is evaluated using the at-lake experimental data.

### 4.1. Tank Experiments

**Figure 3:**
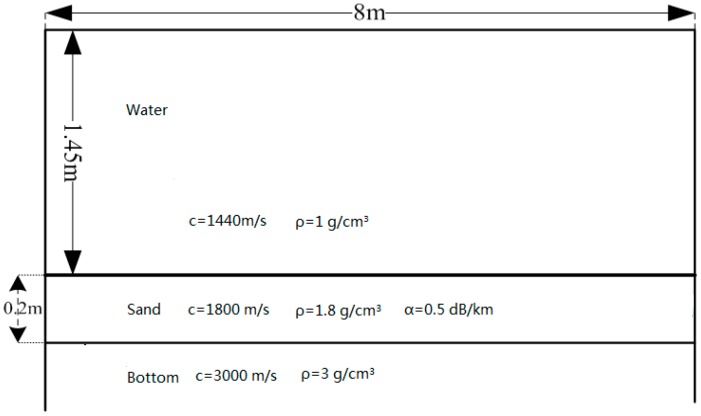
Tank geoacoustic parameters.

The laboratory tank has length of 8 m, width of 4 m, and depth of 2 m. The four walls of the tank are covered by a layer of sound-absorption material. The geoacoustic parameters of the tank are shown in Figure 3.

**Figure 4:**
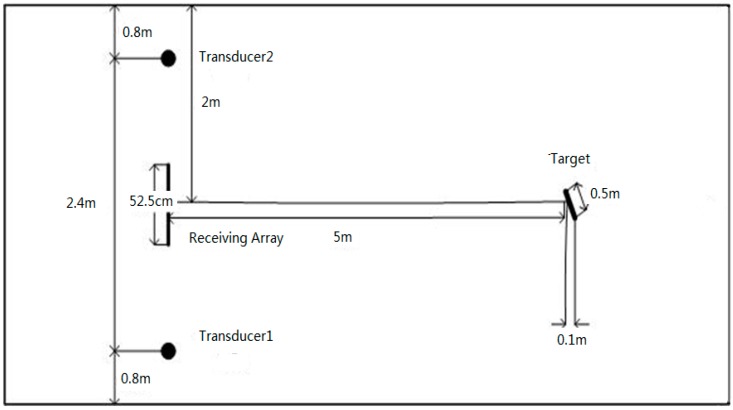
Tank experimental configuration.

Figure 4 shows the tank experimental configuration. The receiving array consists of nine hydrophones with the inter-element spacing of 0.075 m. Two transmitting transducers located at two ends of the receiving array are separated with a distance of 2.4 m. A hollow stainless steel cylinder used as a target has length of 0.5 m, diameter of 0.2 m, and thickness of 0.03 m. The target is placed at a distance of 5 m. Two transducers, the receiving array and the target, are placed at the same depth of 0.5 m below the water surface. To capture the target diversity by the MIMO system, the target is placed at 11.5° from the receiving array horizontal direction. The data collection system has a sampling rate of 48 kHz.

Firstly, the detection performance of the distributed phased-MIMO sonar system is evaluated with 4 ms PCW signals. Transducer 1 transmits 7.5 kHz signal meanwhile Transducer 2 transmits 8.5 kHz signal. For comparison, the phased-array sonar system utilizes Transducer 2 to transmit either 7.5 kHz signal or 8.5 kHz signal. The corresponding signal is amplified with $\sqrt{2}$ times that utilized in the distributed phased-MIMO sonar system for maintaining a constant transmission power.

**Figure 5:**
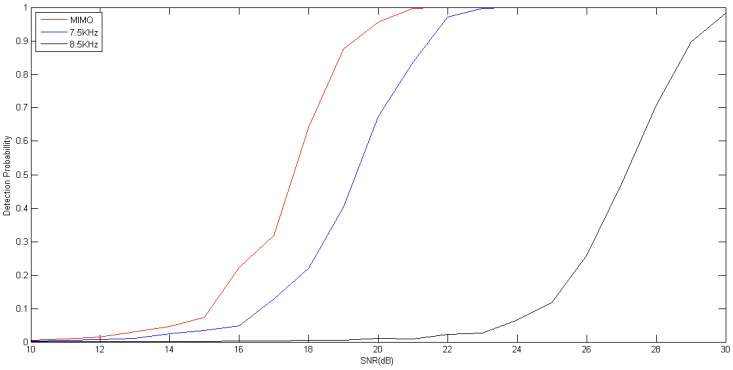
The detection performance of the distributed phased-MIMO sonar system is evaluated using 7.5 kHz and 8.5 kHz PCW signals in comparison with that of the phased-array sonar system. The probability of false alarm is fixed at 0.001.

Figure 5 shows the detection performance of two kinds of sonar systems. The probability of false alarm is fixed at 0.001. It is observed from Figure 5 that the distributed phased-MIMO sonar system requires SNR of 19.5 dB to achieve the probability of detection of 90% while the phased-array sonar system requires SNR of 21.5 dB or 29 dB, respectively, for 7.5 kHz or 8.5 kHz PCW signal. For the case, the distributed phased-MIMO sonar system has captured the target diversity. In addition, the phased-array sonar system performs better with low frequency than with high frequency due to the less acoustic attenuation.

**Figure 6:**
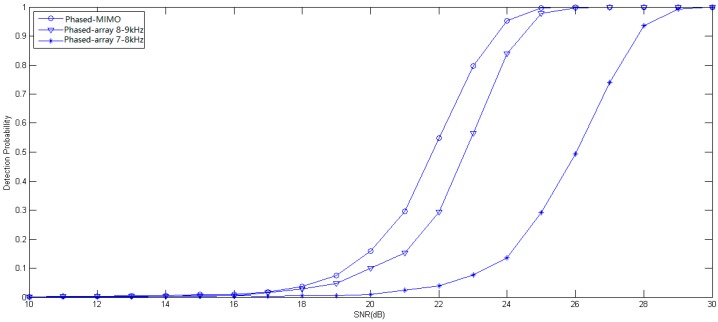
The detection performance of the distributed phased-MIMO sonar system is evaluated using 7–8 kHz and 8–9 kHz LFM signals in comparison with the phased-array sonar system. The probability of false alarm is fixed at 0.001.

Further, the detection performance of the distributed phased-MIMO sonar system is evaluated using the broadband signals, 4 ms LFM signals. Transducer 1 transmits 7–8 kHz signal, meanwhile, Transducer 2 transmits 8–9 kHz signal. For comparison, the phased-array sonar system only uses Transducer 2 to transmit the 7–8 kHz or 8–9 LFM signal. The signal amplitude is $\sqrt{2}$ times that of the phased-MIMO system. Figure 6 shows the receiver performance curves of two detectors with a probability of false alarm of 0.001. From this figure, one can find that the phased-MIMO system requires an SNR of 23.5 dB to achieve a probability of detection of 90% while the phased-array system requires SNR of 24.5 dB or 27 dB respectively corresponding to 7–8 kHz or 8–9 kHz FLM signal. In addition, the distributed phased-MIMO system requires a higher SNR with the broadband signal than with the narrow signal to achieve the same detection performance. It can be seen by comparing Figure 5 with Figure 6 that the detection probability of the narrowband signal looks higher than that of the broadband signal. For the broadband signal, the whole energy is evenly distributed over all frequency bins, thus, there is more propagation loss for high frequency components. Moreover, the frequency diversity results in a low detection probability using the broadband signal.

### 4.2. At-Lake Experiments

**Figure 7:**
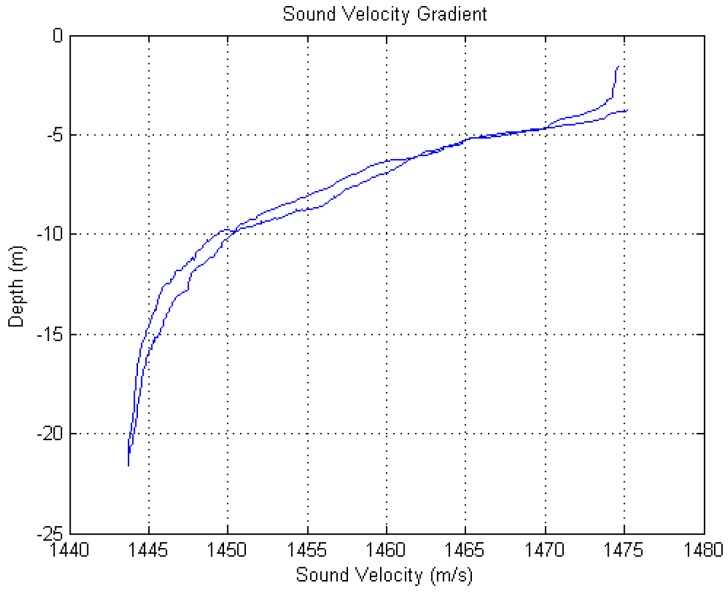
Sound speed profile.

**Figure 8:**
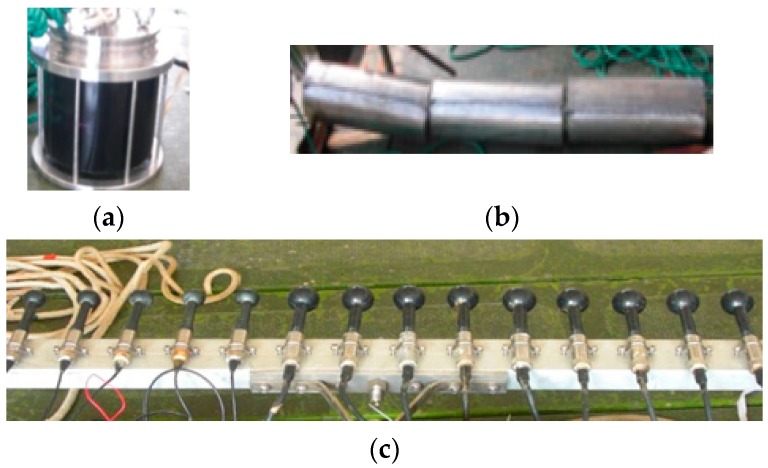
Transducer (**a**); target (**b**); and the receiving array (**c**).

**Figure 9:**
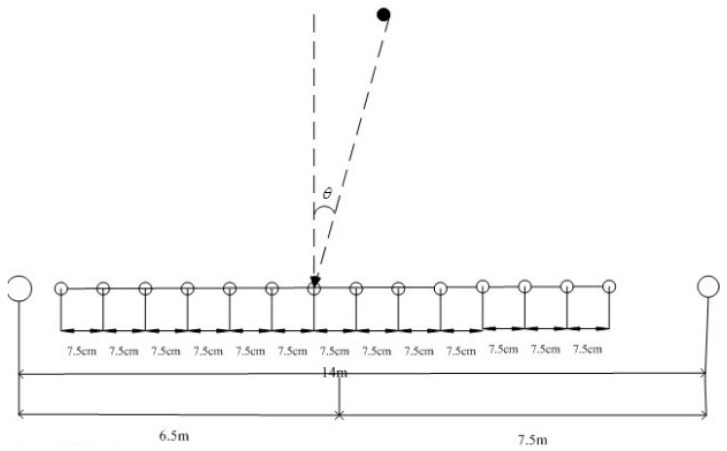
At-lake experimental configuration.

The at-lake experiments were performed during summer in Lake Mogan. The average depth of water in the experimental area is about 23 m. The measurement of the sound speed profile in the experiment is shown in Figure 7. Clearly, it is a negative gradient environment. For evaluating the effectiveness of the signal model of the distributed phased-MIMO sonar, we have designed an experimental system which consists of a 14-element receiving array with the inter-element spacing of 0.075 m and two omnidirectional transmitting transducers with a distance of 14 m as shown in Figure 8. Two transducers are placed at two ends of the receiving array to see different aspects of an extended target. The extended target consists of three hollow stainless steel cylinders as shown in Figure 8, each with a length of 0.5 m, diameter of 0.2 m, and thickness of 0.03 m. Two transducers and the receiving array were placed at a depth of 4 m below the water surface while the extended target was flexibly suspended by two cables from a stationary boat to a depth of 5 m. The distance between the target and the receiving array is about 200 m, measured by a global positioning system (GPS) with a location precision of about 10 m. Figure 9 demonstrates the experimental configuration. The data collection system has a sampling rate of 48 kHz. In the experiments, we choose 20 ms PCW and LFM signals with non-overlap in the frequency domain as the orthogonal waveforms. For comparison, we have also considered a phased-array sonar experimental system which consists of one transducer and the receiving array of the MIMO sonar system.

Firstly, the localization performance of the distributed phased-MIMO sonar system is evaluated with the PCW signal. In the MIMO system, Transducer 1 transmits a 6 kHz signal meanwhile Transducer 2 transmit an 8 kHz signal. The stationary target is at bearing of 0°. Due to the omnidirectional transmission, the target can be simultaneously illuminated by 6 kHz and 8 kHz signals. In the phased-array system, only the 6 kHz signal is transmitted by Transducer 1 to illuminate the target. For maintaining a constant transmission power, the amplitude of the transmitted signal is $\sqrt{2}$ times that utilized in the MIMO system. Due to the failure of two receiving channels, only echoes received by the other 12 channels are utilized for target localization. The experimental system works in the MIMO mode and the phased-array mode by turns. It means that the orthogonal waveforms of the MIMO system and the transmitted waveform of the phased-array system are transmitted in the recycling mode.

**Figure 10:**
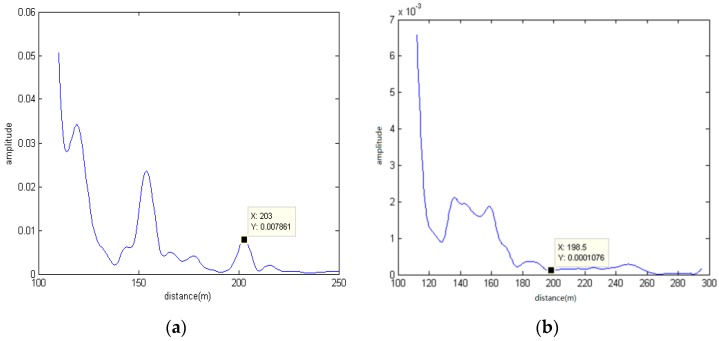
Target range is estimated by two kinds of sonar systems with PCW signals: (**a**) Distributed phased-MIMO sonar system; (**b**) Phased-array sonar system.

Figure 10 demonstrates the results of target localization of two systems. By comparing Figure 10a with Figure 10b, we can see that it has a peak at distance of 203 m for the MIMO sonar system. However, the phased-array sonar system cannot find the target. Considering the location precision of 10 m of the GPS, the target range provided by the MIMO sonar system is close to the true position of the target. For the case, due to being flexibly suspended by two cables from the boat, the target has an unstable posture in water. When the target is illuminated from two angles, the strong target echoes can be received by the MIMO sonar system. For the phased-array sonar system, although Transducer 1 also illuminates the target due to its omnidirectional transmission, the weak echoes result in the impossibility of localization of the target. In a sense, target diversity is helpful for localization of a target in the MIMO sonar system.

**Figure 11:**
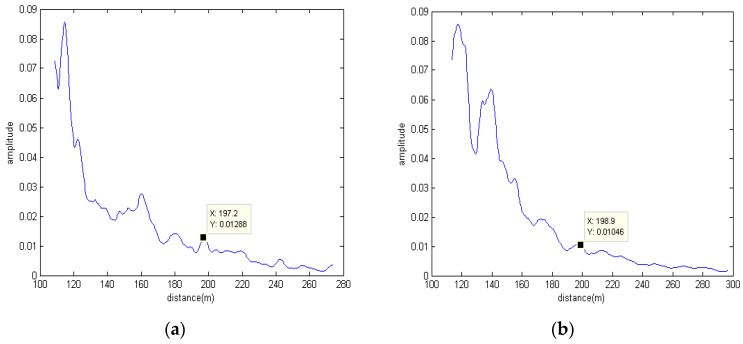
Target range is estimated by two kinds of sonar systems with LFM signals: (**a**) Distributed phased-MIMO sonar system; (**b**) Phased-array sonar system.

Furthermore, we utilize LFM signals to evaluate the localization performance of the distributed phased-MIMO sonar system. Transducer 1 transmits a 6–8 kHz signal while Transducer 2 transmits an 8–10 kHz signal. In the phased-array sonar system, we utilize Transducer 1 to Transmit 6–8 kHz signal. Due to the failure of Channel 5, only echoes received by 13 other channels are utilized for estimating the target distance. For the broadband signal, the beamforming operation in the frequency domain is carried out over the separated target echoes before the RCI operation is utilized for estimating the target range. Figure 11 shows the experimental results with LFM signals. From Figure 11a, we can see that the distributed phased-MIMO sonar system has a peak at a distance of 197.2 m. For the phased-array sonar system, there is also a peak at a distance of 198.9 m. However, the peak value of the latter is lower than that of the distributed phased-MIMO sonar system enjoying target diversity. In addition, due to the frequency diversity and the more high frequency propagation loss, the amplitude of the target-range curve with LFM signals is smaller than that with narrowband signal which can be seen by comparing Figure 10 with Figure 11.

## 5. Conclusions

The paper has presented a joint processing framework for a distributed phased-MIMO sonar system. It combines the diversity gain with beamforming gain to improve the detection capability. The signals with non-overlap in the frequency domain are utilized as MIMO orthogonal waveforms to capture the spatial diversity in the underwater acoustic channels. The beamforming technique is utilized to enhance the weak target echoes due to the omnidirectional transmission. Target range can be calculated using the estimated target bearing, the time-delay estimate provided by the RCI processor, and the geometric parameters of the array configuration. The numerical simulations and the tank experiments have shown that the GLRT detector of the MIMO system outperforms that of the phased-array system in the realm of high SNR. The robustness of the MIMO sonar system has been demonstrated by localization of a target in at-lake experiments.
